# Circulating tumour cells are a prognostic indicator in advanced high-grade serous ovarian cancer and are associated with platelets and immune cells following dissemination

**DOI:** 10.1038/s41416-025-03227-7

**Published:** 2025-10-10

**Authors:** Mark P. Ward, Faye Lewis, Catherine O’Gorman, Lucy A. Norris, Sarah E. Lochrin, Laura E. Kane, Tanya E. Kelly, Bashir M. Mohamed, Ashitha Ramesh, Roisin O’Connor, Elaine Kilgour, Brian Henderson, Marika Kanjuga, Sinead Hurley, Laura Edgerton, Prerna Tewari, Kathy Gately, Lorraine O’Driscoll, Karsten Hokamp, Siobhan Cashman, Gavin McManus, Doug A. Brooks, Stavros Selemidis, Niamh Coleman, John Kennedy, Waseem Kamran, James P. Beirne, Patrick Maguire, Feras Abu Saadeh, Karen Cadoo, Cara M. Martin, John J. O’Leary, Sharon A. O’Toole

**Affiliations:** 1 Molecular Pathology Research Lab, Coombe Women and Infants University Hospital, Dublin, 8 Ireland; 2 Department of Histopathology, Trinity College Dublin, Dublin, Ireland; 3 Trinity St James’s Cancer Institute, St James’s Hospital, Dublin, Ireland; 4 Department of Obstetrics and Gynaecology, Trinity College Dublin, Dublin, Ireland; 5 The Haematology, Oncology and Palliative Care (HOPe) Directorate, St James’s Hospital, Dublin, Ireland; 6 CRUK Manchester Institute Cancer Biomarker Centre, University of Manchester, Manchester, UK; 7 Thoracic Oncology Research Group, Trinity Translational Medicine’s Institute, Trinity College Dublin, St James’s Hospital, Dublin, Ireland; 8 School of Pharmacy and Pharmaceutical Sciences, Trinity Biomedical Sciences Institute, Trinity College Dublin, Dublin, Ireland; 9 Department of Genetics, School of Genetics and Microbiology, Trinity College Dublin, Dublin, Ireland; 10 BD Research Centre Ireland, Limerick, Ireland; 11 Microscopy and Imaging Centre, School of Biochemistry and Immunology, Trinity College Dublin, Dublin, Ireland; 12 Clinical and Health Sciences, University of South Australia, Adelaide, SA Australia; 13 Centre for Respiratory Science and Health, School of Health and Biomedical Sciences, RMIT University, Bundoora, VIC Australia; 14 Division of Gynaecological Oncology, St James’s Hospital, Dublin, Ireland

**Keywords:** Ovarian cancer, Prognostic markers, Tumour biomarkers

## Abstract

**Background:**

Circulating tumour cells (CTCs) are rare yet crucial biomarkers with significant prognostic potential across different cancer types. However, their role in high-grade serous ovarian cancer (HSGC) is not well defined. To capture the full spectrum of CTCs found in HGSC, we employed an EpCAM independent enrichment technique in patients with advanced HGSC and investigated the prognostic value and molecular signatures of these rare cells.

**Methods:**

CTC enumeration was performed in 43 newly diagnosed patients with HGSC using Parsortix® CTC enrichment and benchmarked against a metastatic breast cancer (MBC) cohort for which the device is FDA approved. CTCs were also isolated from the ovarian vein of patients with HGSC during primary cytoreductive surgery. CTCs were assessed as prognostic markers in patients with HGSC. FACS single cell sorting and scRNAseq was performed on CTCs isolated from the ovarian vein.

**Results:**

CTCs isolated using Parsortix® enrichment in HGSC ranged between 1-22 cells/7.5 ml blood. Concordance was seen between Parsortix® enrichment and CellSearch® enumeration in patients with MBC (R^2^ = 0.8786). CTC clusters were isolated from the ovarian vein (*P *= 0.0195) and were cloaked in platelets/immune cells. Detection of CTCs in patients with HGSC was predictive of a poorer progression free survival (*P *= 0.0183). Patients with CTCs were found to have increased serum levels of CD73 (*P *= 0.0311). scRNAseq of CTCs isolated from the ovarian vein identified enrichment in genes associated with immune signalling.

**Conclusions:**

Peripheral CTCs isolated from patients with HGSC were predictors of a poor prognosis. The ovarian vein was found to be a rich source of disseminating CTC clusters in HGSC. Further studies are warranted to investigate the utility of CTCs as markers of neoadjuvant chemotherapy response as well as for longitudinal monitoring. Molecular analysis of CTCs in HGSCs reveals a potential role of the immune system in CTC-mediated haematogenous metastasis.

## Introduction

Circulating tumour cells (CTCs) are rare haematogenous circulating epithelial derived cancer cells that are released into the blood circulation and represent a critical intermediate phase of the metastatic cascade [1]. CTCs are a prognostic indicator in metastatic breast, colorectal and prostate cancers, with higher numbers of CTCs predicting shorter disease-free intervals and overall survival [2–4]. However, CTCs are rare and vary phenotypically with a short half-life in the blood circulatory system, presenting challenges for detection, particularly in cancers not usually associated with haematogenous spread, such as ovarian cancer.

High-grade serous ovarian cancer (HGSC) is the most common ovarian cancer subtype [5–7]. Due to the lack of effective screening tools and its non-specific symptoms, the majority of patients with HGSC present with advanced disease (The International Federation of Gynaecology and Obstetrics (FIGO); FIGO stage III or IV) at diagnosis [8]. The 5-year survival rate for advanced HGSC is poor at <30% [9]. The dissemination of cancer cells from the primary ovarian tumour is complex, with HGSC cell dissemination thought to occur primarily by transcoelomic/intraperitoneal metastatic spread, with cell homing mediated by ERBB3 mechanisms to the omentum and/or peritoneal fluid mechanics [10, 11]. Less is known about the transition of HGSC cells that disseminate through the venous circulatory system, lymphatic mediated metastasis, and nerve-related metastasis [12]. Thus, the area of haematogenous dissemination of HGSC tumours via CTC dissemination remains an understudied area.

The CellSearch® system by Menarini Biosystems is currently the only FDA approved CTC enumeration platform, which captures CTCs using ferromagnetic beads coated with antibodies specific to EpCAM. EpCAM positive CTCs are characterised by; a high nuclear to cytoplasmic ratio, being larger than white blood cells, the presence of cytokeratin (CK) 8, 18, and 19, and the absence of cluster of differentiation 45 (CD45) expression [13]. In ovarian cancers, CellSearch® detects CTCs in only 30% of patients, limiting its utility as a tool for HGSC prognosis [14, 15]. In ovarian cancer, the epithelial marker CK7 can be used in combination with other markers (WT1, PAX8, EpCAM) for molecular phenotyping HGSC in tissue biopsies. This suggests that previous studies using CellSearch® may potentially have underestimated CTC positivity in patients due to the lack of specific marker inclusion. Moreover, CellSearch® is unable to capture CTCs that do not express EpCAM such as cells with a mesenchymal phenotype. Recently the Parsortix® PR1 (ANGLE PLC) received approval from the FDA as a CTC enrichment device for metastatic breast cancer (MBC) [16]. This device is antigen-independent and enriches CTCs based on size and deformability. The ability to enrich the full plethora of CTCs from patients and evaluate their unique clinical utility is essential for CTC enumeration to be standardised for clinical practice.

Immune cells and platelets are important factors used by cancer cells to facilitate CTC release and protection in circulation [17–19], however, little is known about their role in ovarian cancers. A high platelet count is a feature of ovarian cancer, including HGSC [20]. Platelet cloaking of ovarian cancer cells promotes cell invasion and migration by the induction of epithelial to mesenchymal transition (EMT) [21, 22]. Platelet cloaking of CTCs has also been found to impact immune cell recognition of CTCs in the circulation and may have a role in CTC-mediated immune evasion. Platelet cloaking of cancer cells has been implicated in the low detection of CTCs and may impact on current enrichment systems through the downregulation of EpCAM expression. Understanding the interaction between platelets and CTC release into the circulatory system is important to understand the mechanisms of CTC dissemination in HGSC and to understand if the influence of platelets and immune cells occurs at the primary tumour site or within the blood circulation.

This study focuses on the isolation, characterisation and molecular interrogation of CTCs from a single ovarian cancer histological subtype, advanced HGSC. Using an optimised CTC enrichment platform and CTC immunofluorescence staining protocol, the utility of CTC isolation in newly diagnosed HGSC is benchmarked to a known model of high CTC trafficking, MBC, and the prognostic role of CTCs in HGSC investigated. We describe sampling of the ovarian vein for CTC detection and characterisation in patients undergoing primary cytoreductive surgery. We also interrogate the interactions of CTCs with platelets, immune cells and inflammation markers and use single cell RNA sequencing (scRNAseq) of CTCs to begin to better understand the role of CTCs in HGSC.

## Methods

### Patient cohort

Patients were prospectively recruited to this study between January 2020 and August 2023, from St. James’s Hospital, Dublin. All patients included in this study gave full and informed written consent (St. James’ Hospital/Tallaght University Hospital Joint Research Ethics Committee (ID:2095)). Clinicopathological data was collected for each patient included in this study, with Table 1 summarising demographics for patients with HGSC included in this study. Supplementary Table 1 summarises the demographics for patients with MBC. See supplemental methods for details on healthy donor blood used for in vitro experiments in this study. All experiments were performed in accordance with the Helsinki declaration and relevant guidelines and regulations.

**Table 1:** Clinicopathological details of HGSC patient cohort included in this study.

Variable	All patients (*n *= 43)
Median age (Years)	63
Range	(38-83)
Median BMI	25.5
Range	(16-46)
Menopausal status	
Premenopausal	7 (16%)
Postmenopausal	36 (84%)
Treatment type	
Primary cytoreductive surgery	16 (37%)
Neoadjuvant/Interval cytoreduction	13 (30%)
Chemotherapy only	14 (33%)
FIGO stage	
IIIA	2 (4%)
IIIB	3 (7%)
IIIC	21 (49%)
IVA	5 (12%)
IVB	12 (28%)
BRCA status	
Germline	8 (21%)
Somatic	1 (1%)
Negative	29 (76%)
Not tested	3
Awaiting	2

### Blood sampling from peripheral and ovarian vein for CTC isolation

Venous blood was taken into a K2EDTA blood tube for CTC enumeration and processed as described below. Plasma and serum samples were collected, aliquoted and stored at –80 °C until assay analysis. All samples were processed and stored within 2 h of venepuncture. For ovarian vein sampling during primary cytoreductive surgery, the infundibulopelvic ligament/gonadal vessels were mobilised and the ureter was identified before the vessels were clamped proximally, above the pelvic brim, using artery forceps. This approach ensures oncological safety and prevents uncontrolled bleeding once the sampling is finished. It also ensures the blood is aspirated from the venous rather than arterial circulation. A 23-gauge needle attached to a 10 mL syringe was then used to carefully aspirate blood from the ovarian vein prior to transfer to a K2EDTA blood tube.

### Circulating tumour cell isolation and enrichment

For Parsortix® CTC enrichment, all blood samples were collected in 9 mL Vacuette® K3EDTA tubes (Greiner Bio-One) and processed within 4 h of blood draw. Samples were enriched using a 6.5 µm separation cassette, following the manufacturer’s guidelines. Captured cells were enumerated using in-cassette staining on the Parsortix®. Briefly, the Parsortix**®** captured cells were fixed with 4% paraformaldehyde (Sigma Aldrich, Ireland) followed by incubation with permeabilization buffer Inside Perm (Cat:130-090-477, Inside stain Kit; Miltenyi Biotec, Germany). For metastatic breast cancer patient samples, slides were incubated with EpCAM, (Cat: 324210, Alexa Fluor 488, Biolgend; 1:100) Cytokeratin 19 antibody (Cat: ab205445, Alexa Fluor 488, Abcam, Netherlands; 1:100), pan cytokeratin (Cat:130-118-964, FITC, Miltenyi Biotec, Germany; 1:100), HER2 (Cat: 324406, PE, Biolegend; 1:100), CD45 (Cat: 368538, Alex Fluor 647, Biolegend; 1:100) as well as the nucleic acid dye Hoechst 33342 (Thermofisher, Ireland, 1: 120 (v/v))/DAPI (4′,6-diamidino-2-phenylindole, Thermofisher, Ireland). For high grade serous ovarian patient samples, slides were incubated with cytokeratin 7 antibody (Cat: ab185048, Alexa Fluor 488, Abcam, Netherlands; 1:100), pan cytokeratin (Cat:130-118-964, FITC, Miltenyi Biotec, Germany; 1:100), EpCAM, (Cat: 324210, Alexa Fluor 488, Biolgend; 1:100), CD42b (Cat: 303906, PE, Biolegend; 1:100), CD45 (Alex Fluor 647, Biolegend; 1:100) as well as Hoechst 33342 (Thermofisher, Ireland, 1: 120 (v/v))/DAPI (4′,6-diamidino-2-phenylindole, Thermofisher, Ireland). Cells displaying the phenotype of EpCAM/panCK/CK19+ or CK7+ plus DAPI+/CD45− with a round intact morphology were considered CTCs. See supplemental methods for further details on the CTC isolation technologies used in this study.

### Measurement of routine blood, vascular inflammation, and immune homeostasis markers in patients with HGSC

Soluble ICAM-1 (sICAM-1), soluble VCAM-1 (sVCAM-1), and CD73 were measured using commercial duoset ELISA kits (Bio-Techne/R&D Systems, Ireland) as per manufacturer’s instructions. The available results for CA-125, lymphocytes, haematocrit (HCT), haemoglobin (Hb), neutrophils, platelets, and CRP were obtained from the clinical laboratory analysis of patients at diagnosis and used for the correlation with immune variables and CTC counts.

### Platelet and neutrophil isolation and co-culture

Healthy donor whole blood was collected by venipuncture using a 19-gauge butterfly needle without a tourniquet to avoid platelet activation. For the preparation of washed platelets, blood was collected into Acid-Citrate-Dextrose (ACD: 38 mM citric acid, 75 mM sodium citrate, 124 mM D-glucose) which acted as an anticoagulant (15% vol/vol) and centrifuged at 170 × *g* for 10 min. Platelets were counted using the Sysmex XP-300™ Automated Haematology Analyser (Sysmex, Japan) and were co-incubated with SKOV-3 cells for 24 h in a 1000:1 ratio as previous described [21]. For neutrophil isolation, 9 mL of blood was drawn from a healthy donor and isolated as described previously [23].

### FACS sorting of circulating tumour cells and single cell RNA sequencing

ClearCell FX enriched cells were collected and centrifuged at 300 × *g* for 5 min. Cells were resuspended in 300 μL of PBS + 2% BSA. Captured cells were stained with an antibody cocktail including markers of CTC detection (EpCAM, E-Cadherin, EGFR, MUC1, HER2, and N-Cadherin, Becton Dickenson, USA) and immune markers (CD45 and CD66b) (Becton Dickenson, USA). All cell sorting was performed on the BD Melody™ (Becton Dickenson, USA) cell sorter using FACS Chorus software. Briefly, CTCs were gated on single cells, live (7AAD), CD45-negative and EpCAM/E-Cadherin positive cells within this population (See Supplementary Fig. S3 for gating strategy). See supplemental methods for details on single cell RNA sequencing.

### Statistical analysis

All quantified cell line data are presented as the mean ± SD for at least three independent experiments. All patient data are presented as induvial points as box plots of median. Student paired and unpaired t-test analysis, Wilcoxon matched pairs test and analysis of variance (ANOVA) was conducted using Prism 9 (GraphPad Prism, San Diego, California, USA). Spearman R correlations were performed to test strength and direction of association between two ranked variables. One way ANOVA was used for statistical analysis involving three or more groups. Log-rank (Mantel–Cox) survival analysis for progression free survival (PFS) and overall survival (OS) was performed using Prism 9 with all patients having at least 1 year follow up observation time. scRNAseq analysis was performed using R toolkit Seurat. Significance was considered to be *P *< 0.05.

## Results

### Patient characteristics for benchmarking CTC isolation in HGSC

**Fig. 1:**
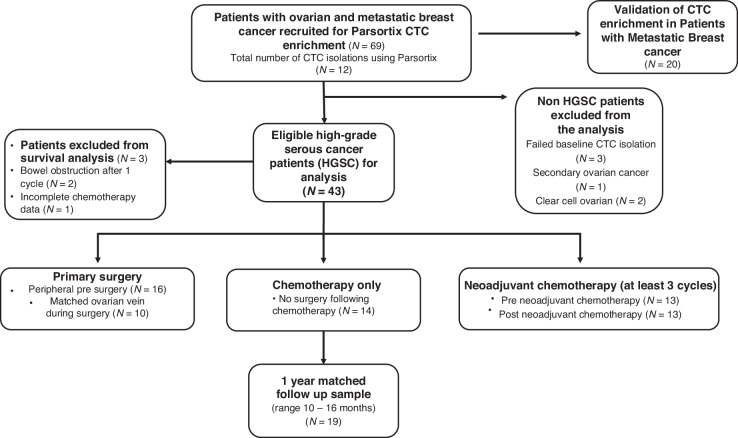
Schematic overview of patient inclusion criteria and study design. All patients included in this study gave informed and written consent.

To assess the utility of the Parsortix® device for the enrichment of CTCs and for optimisation prior to HGSC patient inclusion, 20 MBC patients were recruited for CTC enrichment and enumeration optimisation. Ten MBC patients also had matched sampling performed for direct comparison between Parsortix® isolation/enumeration and CellSearch® CTC enumeration. The MBC cohort was heterogenous in terms of histological subtype, treatment status, and treatment type (Supplementary Table 1) and a single blood sample was obtained at a treatment progression time point. Blood samples from 43 patients with stage III-IV HGSC were included in this study (Fig. 1). The HGSC cohort either had neoadjuvant chemotherapy (30%) followed by interval cytoreduction surgery, primary cytoreduction surgery (37%), or only received chemotherapy following their diagnosis as were not suitable for surgery (33%) (Table 1). Forty HGSC patients were included for survival analysis who met the inclusion criteria of standard of care treatment, with three patients excluded due to treatment complications following 1 cycle of chemotherapy and incomplete neoadjuvant chemotherapy completion data. In total, 112 CTC isolations were performed using Parsortix® in this study (43 HGSC and 20 MBC baseline sampling CTC enrichments), and 42 follow up sample from patients with advanced HGSC (13 matched pre and post neoadjuvant chemotherapy isolations, 10 ovarian vein samples and 19 1-year follow up samples (Fig. 1).

### Benchmarking of the isolation and enumeration of CTCs in a cohort of metastatic breast cancer patients using Parsortix®

**Fig. 2:**
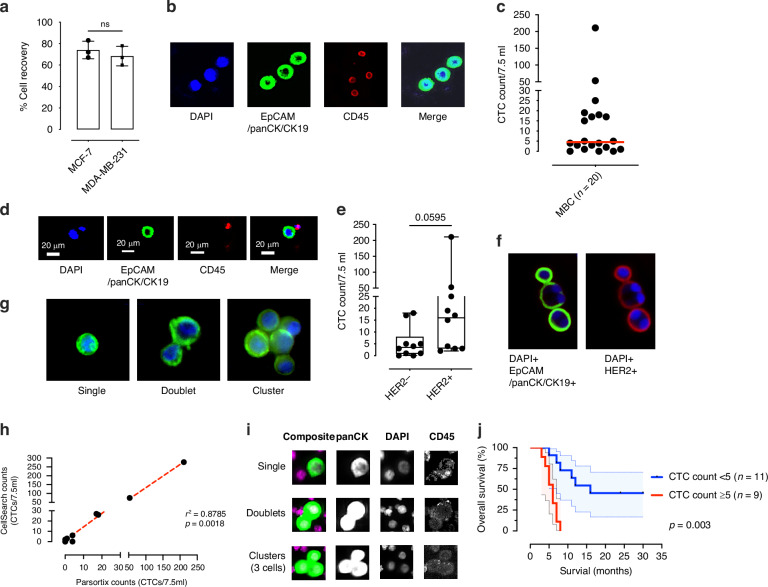
Optimisation of antigen independent enrichment of CTCs and enumeration using a model of high CTC trafficking. **a** 200 cytotracker green labelled MCF-7 cells were spiked into healthy donor blood and enriched using Parsortix® CTC enrichment. Cells were counted in-cassette and post flush from device into a 96 well plate (*n *= 3). **b** Representative image of MCF-7 cells stained with DAPI (blue), EpCAM/panCK/CK19 (green), CD45 (red) and merged image. **c** Numbers of EpCAM/panCK/CK19 + CD45- CTCs captured from *n *= 20 metastatic breast cancer (MBC) patients per 7.5 ml of peripheral blood. **d** Representative images of cells captured from MBC patients. **e** Numbers of detectable CTCs in patients with HER2 positive (*n *= 10) and HER2 negative (*n *= 10) stained CTCs. **f** Representative image of HER2 + CTC cluster isolated using Parsortix®. **g** Images of single, doublet, and CTC clusters in patients with MBC using Parsortix®. **h** Direct comparison of Parsortix enrichment compared to CellSearch® (*n *= 10). **i** CellSearch® representative images of CTC singles, doublets, and clusters identified in MBC patients. **j** OS of patients with MBC following identification of CTCs using Parsortix® (*n *= 20). Significance of *P *< 0.05.

To validate CTC enrichment using Parsortix® and for enumeration efficiency, a breast cancer cell line model was used (MCF-7 and MDA-MB-231) and spiked into healthy blood. Both the EpCAM_high_ (MCF-7) and EpCAM_low_ (MDA-MB-231) expressing cell lines had similar recovery rates of (65–73%) (Fig. 2a). Next, in-cassette staining showed that MCF-7 cell line expressed the epithelial markers EpCAM/panCK and CK19, (Fig. 2b) and lacked the immune marker CD45. Following cell line optimisation, CTC isolation was performed on 20 MBC patients (see Supplementary Table 1 for demographic details) using the Parsortix® and compared with CTC enumeration from the same patients with the CellSearch® system. Using the Parsortix® system, the range of cells isolated was 1–220 CTCs per 7.5 ml of blood (Fig. 2c). CTCs were identified as single cells, cells with CD45+ immune cells attached, doublets, or clusters (3 or more cells) from patients (Fig. 2d, g). CTCs were also stained with the marker HER2, with 50% of MBC patients having at least 1 HER2 + CTC detected, regardless of HER2 tissue status. (Fig. 2e, f). When patients with MBC were benchmarked (*n *= 10) comparing Parsortix® and CellSearch®, concordance was seen in matched samples between the two CTC enrichment devices, (Fig. 2h), (R^2^ = 0.8786). Next, we looked at the effect of Parsortix® derived CTC counts on survival in the MBC cohort. Single, doublets and clusters of CTCs were isolated using CellSearch®. Using CTC cut offs previously established by CellSearch® studies, patients with ≥5 CTCs per 7.5 mL of blood detected using Parsortix® were found to have a shorter OS compared to those with <5 CTCs (Fig. 2j) (*P *= 0.003). Patients with high levels of CTCs (≥5 CTCs) were also found to have a higher platelet lymphocyte ratio (PLR) (*P *= 0.0232) and trend towards a higher neutrophil lymphocyte ratio (NLR) (*P *= 0.072) (Supplementary Fig. S1A–D), while CTC clusters of 30 or more cells were also found enriched from peripheral blood as well as CTC doublets and clusters associated with CD45+ immune cells (Supplementary Fig. S1E, F).

### Isolation and characterisation of circulating tumour cells from patients with HGSC

**Fig. 3:**
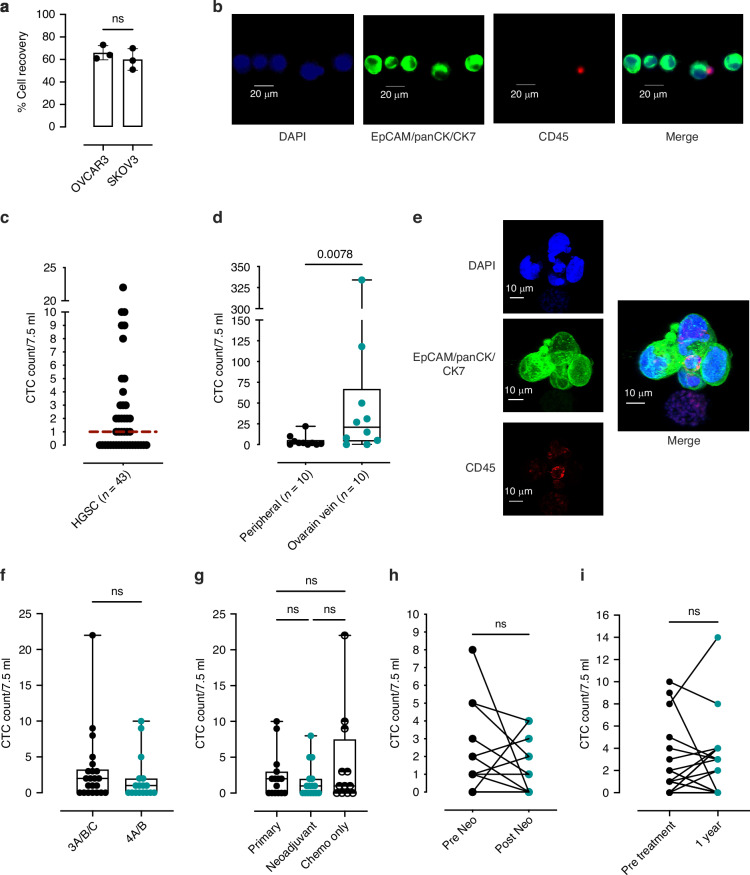
CTCs and CTC clusters can be isolated from peripheral blood and ovarian vein of patients with HGSC throughout their treatment journey. **a** Recovery of the HGSC cell line OVCAR3 and SKOV3 from healthy donor blood (200 cells/7.5 ml blood) stained with HGSC CTC antibody cocktail using Parsortix® (*n *= 3). **b** Representative images of captured OVCAR3 cells with HGSC CTC antibody detection antibodies spiked into healthy donor blood for CTC enumeration and microscopy optimisation. **c** Range of CTCs (CK7/panCK/EpCAM+ CD45- cells) per 7.5 ml blood detected in peripheral blood of patients with newly diagnosed HGSC (*n *= 43). **d** Range of CTCs (CK7/panCK/EpCAM+ CD45-) detected in ovarian vein blood compared to matched peripheral blood samples from patients with HGSC undergoing primary surgery (*n *= 10). **e** Representative confocal images of HGSC ovarian vein isolated CTC cluster interacting with a CD45 positive immune cell. **f** CTC count in patients with HGSC stratified by FIGO staging. **g** Range of CTCs detected in patients stratified by treatment. **h** Range of CTCs detected in patients with HGSC pre and post neoadjuvant chemotherapy. **i** Range of CTCs (1–14 cells (CK7/panCK/EpCAM+ CD45-)) per 7.5 ml blood detected in HGSC patient’s pre-treatment and 1 year post treatment sample point. Significance of *P *< 0.05.

Following validation of the performance of Parsortix® CTC enrichment in patients with MBC, patients with HGSC, (a low CTC trafficking model) were assessed following antibody optimisation. First, cell recovery using Parsortix® CTC enrichment was assessed using ovarian cancer cell lines OVCAR3 and SKOV3, spiked into healthy donor blood. The recovery rate was found to be similar to that of the breast cancer cell lines at 62% (Fig. 3a). Staining with markers of CTC identification EpCAM, panCK and the inclusion of CK7 showed positive staining in OVCAR3 cells (Fig. 3b). Following optimisation, CTC enrichment was performed on blood samples obtained from 43 newly diagnosed patients with confirmed advanced HGSC (Table 1 for demographics). The range of CTCs detected at baseline was (1–22 CTCs per 7.5 ml) with a median of 1 cell per 7.5 ml blood (Fig. 3c) detected in the cohort (*n *= 43).

In a subset of patients who had primary cytoreductive surgery, matched blood samples (*n *= 10) were obtained from the ovarian vein during cytoreductive surgery. CTC counts from the ovarian vein were significantly higher than CTC counts in the matched peripheral blood samples (*P *= 0.0195) Fig. 3d) with up to 334 cells (range 1–334 CTCs) being detected in a single blood sample.

Enriched ovarian vein CTCs were characterised by confocal microscopy, with clusters (80%), being found to be associated with CD45+ immune cells, similar to those found in large MBC clusters (Fig. 3e). 3D confocal imaging revealed that CD45+ cells were embedded in HGSC CTC clusters disseminating from the ovarian vein (Supplementary Video 1 and Supplementary Fig. S1I).

There was no significant difference in CTC count in HGSC patients with Stage III and Stage IV disease (Fig. 3f). There was no difference in baseline CTC counts between patients who underwent primary surgery compared to those who had neoadjuvant chemotherapy followed by interval cytoreductive surgery or those who only had chemotherapy (Fig. 3g). Similarly in matched patient samples, there was no significant difference in CTC median counts pre and post 3-6 cycles of neoadjuvant chemotherapy (*n *= 13) (Fig. 3h). CTCs were detected in patients 1-year post HGSC diagnosis (*n *= 19) (Fig. 3i).

### CTCs as a prognostic marker in patients with advanced HGSC

**Fig. 4:**
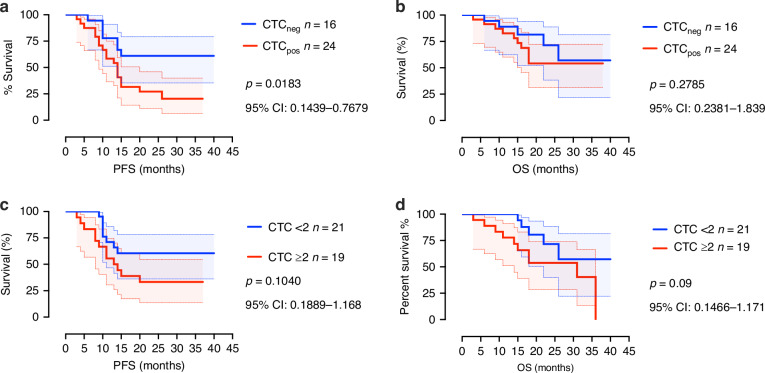
CTCs isolated from patients with HGSC prior to treatment are prognostic and associated with shorter progression free survival. **a** PFS of patients with HGSC who at baseline CTC are negative (*n* = 16) and CTC positive (*n* = 24) (P0.0183). **b** OS of patients with HGSC who baseline CTC are negative (*n *= 16) compared to CTC positive (*n *= 24) (*P *= 0.2785). **c** PFS of patients with HGSC stratified by <2 (*n *= 21) and ≥2 CTCs (*n *= 19) detected. **d** OS of patients with HGSC stratified by stratified by <2 (*n *= 21) and ≥2 CTCs (*n *= 19) detected. Significance of *P *< 0.05.

HGSC patients who were positive for a least one CTC had significantly shorter PFS (*n *= 24) when compared with those who were CTC negative (*n *= 16) (*P *= 0.0183, 95% CI: 0.1439–0.07679) (Fig. 4a). OS was not significantly influenced by CTC count (*P *= 0.2785; 95% CI: 0.2381–1.839) (Fig. 4b) in patients with advanced HGSC. Next, we applied a cut-off of ≥2 CTCs as previously described by CellSearch® in ovarian cancer studies, where no association with PFS (Fig. 4c) or OS (Fig. 4d) was observed.

### Influence of platelets and immune factors on HGSC, CTCs and CTC dissemination

**Fig. 5:**
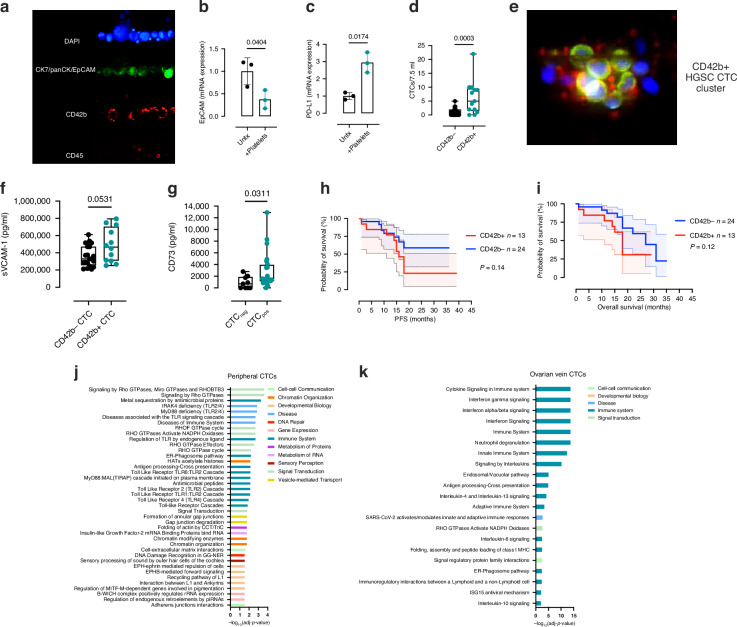
CTCs isolated from blood can be cloaked in platelets, have altered expression of immune checkpoint markers, with ovarian vein CTCs having gene expression profiles associated with altered immune signalling and neutrophil degranulation. **a** OVCAR3 cells spiked into whole blood prior to isolation using Parsortix® are cloaked with CD42b+ positive platelets (*n *= 3). **b** Ovarian cell line mRNA expression of EpCAM and **c** PD-L1 following co-culture with platelets for 24 h. **d** CTC count of CD42b+ cloaked peripheral cells isolated from HGSC patients at baseline. **e** HGSC CTC cluster isolated from the ovarian vein can be cloaked with CD42b+ platelets. **f** Serum sVCAM-1 expression in patients with CD42b- and CD42b+ cloaked CTCs in HGSC (*n *= 33). **g** Serum CD73 expression in CTC negative and CTC positive HGSC patients (*n *= 34). **h** Effect of platelet cloaking of CTCs on the prognostic significance of CTCs ability to predict PFS in HGSC (*n *= 37). **i** Effect of platelet cloaking of CTCs on the prognostic significance of CTCs ability to predict OS in HGSC (*n *= 37). **j** scRNAseq Reactome gene pathway analysis of statistically enriched pathway in peripheral isolated CTCs. **k** scRNAseq Reactome gene pathway analysis of ovarian vein isolated CTCs genes. Significance of *P *< 0.05.

To investigate whether platelets were a factor in CTC detection in HGSC, cells from an ovarian cancer cell line (OVCAR3) were spiked into blood prior to CTC enrichment by Parsortix®. Enriched OVCAR3 cells were found to be cloaked with the platelet marker CD42b (Fig. 5a), indicating that platelets interacted with ovarian cells isolated using Parsortix®. When ovarian cancer cells were co-incubated with platelets for 24 h, EpCAM mRNA expression decreased (*P *= 0.0404) (Fig. 5b) while the mRNA expression of the immune checkpoint marker PD-L1 (CD274) increased (*P *= 0.0174) in cells cloaked with platelets (Fig. 5c). When the platelet marker CD42b was included as a marker in the Parsortix® enumeration immunofluorescence assay, it was noted that patients were found to have a higher number of platelet cloaked CTCs overall (CD42b+ve CTCs) when compared to negative platelet cloaked CTCs (*P *= 0.0003) (CD42b-ve cells), with CTC clusters disseminating in the ovarian vein also being cloaked with platelets (Fig. 5d, e).

Next, we investigated whether CTC counts were correlated with platelet and immune cell counts performed routinely at the same sampling point. CTC count had a weak correlation with HCT and an inverse correlation with neutrophil count (*n *= 34) (Supplementary Fig. S2A) in HGSC. No correlation was found with platelet count, neutrophil to lymphocyte ratio, white cell counts or any of the other routine blood markers.

No association was found between soluble serum VCAM-1 expression and CTC count in patients with HGSC (*P *= 0.0531) (Fig. 5f). No association was found between soluble serum ICAM-1 and CTC status (Supplementary Fig. S2B). However, an increase in the serum expression of the immune checkpoint and homeostasis marker CD73 was found in patients (*n *= 34) who were CTC positive compared to CTC negative (*P *= 0.0311) (Fig. 5g). To determine whether addition of CD42b status on CTCs could improve its prognostic ability, the effect CD42b+ve CTCs on PFS and OS was determined. There was no difference in PFS and OS in patients who were stratified as CD42b+ CTC+ compared to those who were CD42b- (Fig. 5h, i).

Finally, to determine what the potential role platelets and or immune cells have on CTCs released into the circulation from HGSC tumours, 19 CD45-, EpCAM/E-Cadherin+ cells were identified for single cell RNA sequencing (scRNAseq) analysis from 2 patients with matched ovarian vein and peripheral CTC isolations (Supplementary Fig. S3A). CTCs were isolated from the ovarian vein using the Clearbridge FX system rather than Parsortix® due to the high numbers of contaminating immune cells, which were required for FACS cell sorting. The population of CTCs identified was less than 0.1% of the total population of CD45+ immune cells, with approximately 70% of all CD45+ cells isolated from the ovarian vein blood staining for the neutrophil marker CD66b+. Enriched CTCs were FACS sorted based on CD45-, EpCAM/E-Cadherin+ and single cell morphology. Following sequencing, genes associated with both CTC detection (cytokeratins, EpCAM) were detected in CTC FACS sorted cells. scRNAseq of peripheral CTCs from a HGSC patient revealed that genes involved with signalling by Rho-GTPases, MyD88 signalling, and RAS signalling were significantly enriched (Fig. 5j). The scRNAseq dataset revealed that most of the genes enriched in the ovarian vein CTCs were however associated with immune signalling and in particular, immune cytokine signalling, interleukin-6 signalling and neutrophil degranulation (Fig. 5k). We also investigated whether a neutrophil gene signature that we observed in our ovarian vein isolated CTC scRNAseq data could affect ovarian CTCs in vitro. The induction of degranulation and production of neutrophil extracellular traps from healthy neutrophils promoted ovarian cell line proliferation (Supplementary Fig. S4).

## Discussion

Liquid biopsies offer the potential for real-time tumour monitoring of cancer patients using CTC enumeration, circulating tumour DNA (ctDNA) and extracellular vesicle (EV) analysis. However, to have a real impact on the clinical management of patients with ovarian cancer, there is a need to understand how CTCs interact to form homotypic and heterotypic clusters, how they evade the immune system and to define the molecular/cell biology that determines their metastatic potential. CTC enrichment technologies have enormous potential for assessing prognosis, disease monitoring and assessing therapeutic efficacy in patients with ovarian cancer. As a critical first step in this process, we present data focusing on CTCs enriched from both the peripheral venous system and ovarian vein in a group of newly diagnosed patients with advanced HGSC and investigate the influence platelets and immune markers have on CTCs and their prognostic potential.

The clinical utility of CTCs in breast, prostate, and colorectal cancers has been recognised as a marker of adverse disease outcome [24–26]. In contrast, the clinical significance of these rare cells in HGSC is still being established. Ovarian cancers are a heterogenous mix of different cancer types, with HGSC being the most common. Initial ovarian cancer CTC studies classify ovarian cancers together with a mix of histological and molecular subtypes. Using CellSearch® enumeration, many of these studies report that <30% of patient samples were found to be CTC positive [27, 28]. Here, we have investigated HGSC, the most common ovarian cancer subtype, where 70% of all patients present with advanced disease. Using the Parsortix® enrichment method for CTC isolation and an optimised antibody staining panel, we detected at least 1 CTC in 59% of newly diagnosed patients with advanced HGSC, at baseline blood sampling. Our cohort of patients were homogenous, with all patients included having a diagnosis of advanced HGSC. Using this same approach in the well-characterised model of MBC, we detected CTCs in 85% of patients, with excellent concordance to CellSearch® CTC enumeration. Our results are similar to that of smaller study of 16 patients with ovarian cancer, predominately HGSC patients: but using a mixed population of newly diagnosed and recurrent patients with HGSC [29]. In addition to this, a subsequent study by the same group using single cell low pass DNA sequencing of CTCs isolated using Parsortix®, found that 2 out of 3 CK/EpCAM positive CTCs isolated from patients with HGSC using Parsortix® had chromosomal instability identified by the single cell low pass sequencing [30]. However, normal copy number analysis (CNA) profiles were found in cells previously called CTCs, highlighting the need for confirmatory molecular analysis of cells isolated from patients with HGSC. A larger cohort is presented in our study with longitudinal CTC analysis of 43 patients with HGSC to demonstrate consistent CTC isolation with Parsortix® technology. However, a limitation of our study is that molecular interrogation of these cells is needed at the single cell level to complete the evaluation of CTCs in HGSC.

Using Parsortix®, CTCs were detected post chemotherapy and at 1-year follow up in our patient cohort suggesting that CTCs may present a mechanism to quantify and define residual disease in HGSC. However, no reduction in CTC counts was observed in our study post-chemotherapy, which may question the use of just CTC counts as a marker of treatment response in HGSC. Our study did not have sufficient statistical power to determine the association between residual disease, treatment response and CTC count and further adequately powered studies and clinical trials are required to confirm this. Previous studies in early-stage breast cancers treated with neoadjuvant chemotherapy have reported that CTC counts drop following chemotherapy [31], and showed that the presence of one or more CTCs after neoadjuvant chemotherapy predicts relapse and survival in nonmetastatic triple negative breast cancer [32]. Other studies have found patients with a complete response to neoadjuvant chemotherapy still had CTCs present post treatment, suggesting that CTC enumeration alone is not sufficient to aid surgery stratification [33]. However, while tumour reduction may be achieved by chemotherapy in patients with HGSC, the continued release of CTCs may be more indicative of the aggressive nature of the cancer and the ability to disseminate cancer cells from small amounts of the primary tumour.

We report that the ovarian vein is a substantive source of perioperative CTCs in HGSC. In 10 patients who underwent primary cytoreductive surgery, a greater number of CTCs and CTC clusters were found in blood from the ovarian vein compared to the corresponding peripheral blood sample, confirming the release of large numbers of these potentially metastatic cells from the primary tumour into the blood circulation. The right ovarian vein typically drains into the inferior vena cava, while the left ovarian vein terminates in the left renal vein with the lymphatic drainage pathways of the ovaries established via the infundibulopelvic ligament and the ovarian ligament, as well as through the round ligament of the uterus [34]. A previous study in early endometrial cancer patients found that CTCs can be detected from the ovarian vein using CellSearch® in 8 out 10 patients sampled, suggesting that the release of CTCs into the ovarian vein occurs relatively early in endometrial cancer [35]. The latter study found no clinical associations with PFS or OS and CTC detection using CTCs isolated from the ovarian vein in endometrial cancer, whereas we observed an association with PFS using peripheral CTCs. We did not assess the clinical utility of ovarian vein isolated CTCs in our study due to the small number of patients who had ovarian vein sampling performed. However, we report that 80% of CTCs isolated from the ovarian vein were clusters of CTCs, and similar to MBC clusters, were found associated with CD45+ immune cells. Some of these clusters of cells were alarmingly large, >10 CTCs per cluster, which may mean they are most likely already filtered out before they reach the distal peripheral circulation. As clusters are the oligometastatic precursor cells in the metastatic cascade [36–38], we speculate that the release of these HGSC CTC clusters into the ovarian vein occurs relatively early in the disease process, as previously reported in a non-HGSC cohort of patients [39]. However, sampling from the ovarian vein is not a routine procedure and is not always feasible during cytoreductive surgery. Ovarian vein sampling can be performed during primary and interval cytoreductive surgery, if deemed suitable by the surgeon and thus is not a readily available source of CTCs from patients with HGSC. The number of cells within a cluster may dictate the lifecycle of the CTC and its metastatic potential [40], however, further studies are needed to confirm this in ovarian cancer. This process could be an inherent event of cell shedding from the primary tumour or an orchestrated event involving necrosis or highly migratory tumour cells. It will be important to establish whether the relatively rare CTCs observed in peripheral circulation in HGSC have prognostic significance in other ovarian histologic subtypes such as clear cell cancer of the ovary. It is possible that they are just an “artefact” of disease burden in HGSC or that the real culprits for metastasis are lodging well before they reach the circulatory system. Further studies are needed to investigate the lifecycle of CTC clusters isolated from the ovarian vein. CTCs in the peripheral circulation may still be reflective of the programming in the primary or metastasising tumour and co-analysis of the CTCs may be needed to define the cancer biology and prognostic potential.

Cut off values for CTC positivity vary between cancer sites. Previous work using CellSearch® demonstrated that a cut-off for CTC enumeration can be as little as five cells per 7.5 mL of blood for MBC and prostate cancer, and three cells per 7.5 mL of blood for colorectal cancers. In previous CellSearch® studies of ovarian cancer, the positivity rate for CTCs and the numbers of CTCs detected per mL has been relatively low. A prospective multicentre trial including 495 patients with primary ovarian cancer has reported a prevalence rate of disseminated tumour cells of 27% based on a cut-off of 1 CTC [41], with other studies suggesting a CTC cut-off between one and two cells per 7.5 mL of blood in newly diagnosed and recurrent ovarian cancer patients [42]. However, despite this low detection rate CTCs have been found to be a predictor of a poorer PFS and OS in patients with relapsed/recurrent advanced ovarian cancer [43]. In our cohort of HGSC patients, the presence of 1 CTC was a significant cut-off for poor prognosis. We found that patients who had one or more CTCs had shorter PFS compared to those where no CTCs were identified, however the presence of CTCs enriched using the Parsortix® did not predict a poorer OS in our study cohort. Further follow-up is on-going. CA-125 is a well-established diagnostic and prognostic marker for ovarian cancer, however, using a cut-off of 1 CTC per 7.5 ml/blood, there were no relationship with CA-125 levels in our study cohort. In contrast at a cut-off of 2 CTCs, higher CA-125 levels were found in patients who were CTC positive.

The effect of CTC clusters on PFS and OS in our cohort was limited due to small numbers - only 2/43 peripheral blood samples were found to have CTC clusters in our study. Patients with CTC-clusters in other cancer types have been found to have a higher rate of metastasis compared to those with only single CTCs and are a marker of adverse prognosis [44–46]. Patients who were found to be CTC positive had altered levels of haematocrit and haemoglobin, suggesting that patients in whom CTCs are identified may have an altered blood viscosity. Patients with HGSC have an increased procoagulant state [47], and we speculate that CTCs may have a role in this. We investigated whether CTC positivity was associated with increased levels of markers associated with vascular adhesion and invasion which play a role in EMT but found no association with either ICAM-1 or VCAM-1 expression. Previous studies have shown that these markers are central to the adhesion of cancer cells to the vascular endothelium and are involved in the migration of CTCs [48, 49]. CTCs play a role in the interactions between immune cells in breast cancer, especially with neutrophils. We did, however, find increased expression of the immune homeostasis marker CD73. Alongside CD39, the enzymatic activities of CD73 play an essential role in orchestrating the purinergic signals delivered to immune cells through the conversion of ADP/ATP to AMP and AMP to adenosine, driving a shift an ATP-driven proinflammatory environment to an anti-inflammatory milieu induced by the increased levels of converted adenosine [50, 51]. We postulate that in HGSC patients, increased CD73 cells drives an immunosuppressive phenotype, like that seen in the primary tumour, that allows for an increase in CTC circulation through the interaction with platelets [52]. Previously it has been shown that cancer cells cloaked with platelets have an increased CD39 and CD73 expression as well as altered immune evasion [21, 53, 54]. Further work is needed to delineate the role of CD73 in HGSC mediated haematogenous metastasis and whether CD73 is involved in the release of CTCs from HGSC tumours.

Using single cell RNA sequencing of CTCs from the ovarian vein compared to peripheral CTCs, CTCs isolated from the ovarian vein were found to be enriched in pathways associated with immune signalling interleukin-6 signalling (IL-6) and neutrophil degranulation. In previous studies in breast cancer CTCs, over expression of IL-6 and IL-1β or both in CTC-associated neutrophils was sufficient to confer proliferative advantage to breast cancer cells upon dissemination, leading to faster metastasis development and shorter overall survival in mice [48]. Whether these enriched pathways are as result of the utilisation of these pathways by HGSC CTCs or whether the immune cell signature is altered in these samples requires further investigation. However, we postulated that HGSC CTCs may utilise neutrophils when leaving the primary tumour to enter the blood circulation, increasing cell proliferation and increasing their ability to evade immune detection, similar to findings reported in breast cancer.

In conclusion, CTCs can be isolated from patients with HGSC, a traditionally low CTC trafficking model. CTCs are predictors of a poor prognosis in patients with HGSC, with the ovarian vein being a novel and rich source of CTC clusters. This study is the first to describe the shedding of CTCs into the ovarian vein in HGSC, with further studies needed to dissect the life cycle of these cells. The molecular characterisation of ovarian vein CTC clusters is required to identify the potential cellular mechanisms of platelet and immune cell mediated dissemination in HGSC. Further studies are warranted to investigate the utility of CTCs as markers of neoadjuvant chemotherapy response as well as for longitudinal monitoring. Clinical trials are needed to assess the utility of using Parsortix® as a longitudinal monitoring tool in HGSC as well as characterisation of the role CTCs play in organotrophic metastasis in HGSC.

## Supplementary information

3D confocal of CTC cluster isolated from the ovarian vein

Supplemental Methods

Supplemental Figures

Supplemental Table 1

Reactome gene lists

Video 1

## Data Availability

All data generated or analysed during this study are included in this article (and its supplementary information files).
