# Supplementary material for: Circulating tumour cells are a prognostic indicator in advanced high-grade serous ovarian cancer and are associated with platelets and immune cells following dissemination
Source: Br J Cancer. 2025 Oct 10;134(1):22–32. doi: 10.1038/s41416-025-03227-7 (PMC12764790; doi:10.1038/s41416-025-03227-7)
Supplement: Supplementary file 2 — Supplemental Methods [file 41416_2025_3227_MOESM2_ESM.docx]

**Supplemental Methods**

**Patient and healthy donor details.**

Healthy donors were recruited for cell spike-in experiments. All healthy donor participants gave full and informed written consent, and the study had the approval of St. James’s Hospital, and Adelaide and Meath Hospital, Dublin, incorporating the National Children’s Hospital Research Ethics Committee (2012/11/04) and the Coombe Women & Infants University Hospital ethics committee. Multiple CTC sample points were investigated, with patients followed up for at least 1 year post diagnosis. All patients had baseline CTC testing performed in this study. Patients with HGSC undergoing neoadjuvant chemotherapy had pre and post therapy CTC enumeration, while a subset of patients undergoing primary cytoreductive surgery also had ovarian vein CTC enumeration. Observation time for survival analysis was at least 12 months. Details regarding benchmark CTC cohort of patients with MBC are summarised in supplemental Table 1.

**Cell culture.**

Breast (MCF-7, MDA-MB-231) and ovarian (OVCAR-3, SKOV3) cell lines were used for optimisation experiments. MCF-7 and MDA-MB-231 were obtained from the ECACC (UK), with OVCAR-3 and SKOV3 cells were purchased from ATCC (USA). Cells were cultured in complete cell culture media (MCF-7; DMEM media, MDA-MB-231; DMEM media, OVCAR-3; RPMI media, SKOV3; McCoy’s media) supplemented with 10% foetal bovine serum (Sigma Aldrich, Ireland), and 1% Streptomycin antibiotic (Sigma Aldrich, Ireland) and maintained under standard conditions in humidified incubators at 37°C with 5% CO_2_; in cell culture flasks. For routine passaging and spike in experiments, cells were washed with PBS and treated with 1X TrypLE (Gibco, UK). All cell lines were routinely mycoplasma tested.

**Optimisation of Parsortix for CTC imaging and identification.**

Briefly, CFMDA labelled cells were counted and were spiked (200-500 cells) into 7.5 mL of healthy donor blood. Cells were counted in the cassette and recovery efficiency calculated. For evaluation of in-cassette staining, unlabelled breast and ovarian cell lines were spiked into 7.5 mL of healthy donor blood and enriched, as above. Briefly, the captured cells in the cassette were fixed with 4% paraformaldehyde (Sigma Aldrich, Ireland) followed by incubation with permeabilization buffer Inside Perm (#130-090-477, Inside stain Kit; Miltenyi Biotec, Germany). For breast cell lines, samples were incubated with Cytokeratin 19 antibody (Alexa Fluor 488, Abcam, Netherlands; 1:100), pan cytokeratin (Alexa Fluor 488, Miltenyi Biotec, Germany; 1:100), HER2 (PE, Biolegend; 1:100), CD45 (Alex Fluor 647, Biolegend; 1:100) as well as Hoechst 33342 dye (1: 120 (v/v).) For ovarian cancer cell lines, samples were incubated with cytokeratin 7 antibody (Alexa Fluor 488, Abcam, Netherlands; 1:100), pan cytokeratin (Alexa Fluor 488, Miltenyi Biotec, Germany; 1:100), CD42b (PE, Biolegend; 1:100), CD45 (Alex Fluor 647, Biolegend; 1:100) as well as Hoechst 33342 dye (1: 120 (v/v).) Cells were enumerated and imaged using a Cell Imaging System (EVOS® FL) for subsequent analysis and quantified with Image J (National Institute for Health). For more detailed imaging of captured cells, a Leica SP8 scanning confocal microscope equipped with Leica LAS X software was used. For each sample, 100-150 Z-stack steps were acquired from the area of interest, to visualise the three-dimensional (3D) structure of the captured cells. The images were captured in a 2048 x 2048-pixel format. Briefly, for 3D rendering, IMARIS 9 software was used with *Z*-stacks opened in IMARIS in their native format with *Z*-stacks automatically reconstructed into a multi-channel 3D model.

**Real time PCR following co-culture with healthy donor platelets and neutrophils.**

SKOV-3 cells were cultured in 24-well plates (4 × 10^4^ cells per well) for 24 h. Following co-culture with or without platelets and neutrophils for 24 h, cells were washed briefly in PBS, trypsinized using TrypLE and centrifuged to remove the supernatant. RNA was extracted using RNeasy mini kit (Qiagen Ltd., West Sussex, UK) according to the manufacturer's protocol. Total RNA was reverse transcribed to single strand cDNA using the High-Capacity cDNA Archive Kit (Applied Biosystems, Foster City, CA, USA). EpCAM (EpCAM, Hs00901885_m1), PD-L1 (CD274, Hs00204257_m1) and GAPDH (reference gene, Hs02786624_g1) mRNA expression levels were evaluated using commercially by TaqMan RT-PCR (Applied Biosystems, CA, USA) according to the manufacturer's protocol. Results were expressed as the fold change of target gene cDNA relative to untreated control.

**CTC enrichment for CTC enumeration using CellSearch® enumeration and CTC isolation for scRNAseq using ClearCell® FX1.**

For CellSearch® experiments, an additional whole blood sample from 10 MBC patients were collected in CellSave preservation tubes (Menarini Silicon Biosystems, Italy) and processed within 72 h of blood draw. 5-7.5 mL of whole blood was processed on the CellSearch® Autoprep system using the CellSearch® CTC kit (Menarini Silicon Biosystems, Italy), analysed on the CellSearch® Analyzer, and assessed for the presence of CTCs. In all cases, cells displaying the phenotype of EpCAM+/CK+/DAPI+/CD45− cells with a round/oval morphology were classified as CTCs. For single cell RNAseq experiments, blood samples were processed using the ClearCell® FX1 system (Biolidics), as indicated by the manufacturer protocols. 7.5 mL of blood was lysed by adding 22.5 mL of red blood cell lysis buffer, followed by a 10 min incubation and subsequent centrifugation at 500 g for 10 mins. Cells were resuspended in 4 mL of ClearCell® FX1 system Resuspension Buffer and loaded into the ClearCell® FX1 system for CTC isolation on a primed spiral chip. Enriched CTCs and immune cells were then processed for FACS cell sorting using BD Melody.

**Platelet and neutrophil isolation.**

PRP was acidified to pH 6.5 with ACD, and PGE1 (1 µM) was added to avoid platelet activation during centrifugation. Platelets were pelleted by centrifugation at 720 g for 10 min. The supernatant was removed, and the platelet pellet was resuspended in JNL buffer [130 mM NaCl, 10 mM sodium citrate, 9 mM NaHCO3, 6 mM D-glucose, and 0.9 mM MgCl2, 0.81 mM KH2PO4, and 10 mM Tris, pH 7.4] and supplemented with 1.8 mM CaCl2. All platelet samples were processed within 60 min of venipuncture. For healthy donor neutrophil isolation, the blood was diluted 1:1 with Hank’s Balanced Salt Solution (HBSS) and carefully layered over Lymphoprep™ solution (Sigma-Aldrich, MO United States. Enriched granulocyte/neutrophil cells were then re-suspended in the required volume of RPMI-1640 Medium supplemented with 10% (v/v) Foetal Bovine Serum (FBS) and 1% (v/v) penicillin-streptomycin for use. For cancer cell co-culture experiments, a 1:1 ratio of cancer cells to neutrophils was used.

**Single cell RNA sequencing.**

Following CTC identification using FACS Melody, cells from individual samples were sorted using plate holder apparatus for the Melody and a 96-well cooling block (BioCision CoolRack®, San Rafael, CA), which could hold BD Precise™ Single Cell Encoding 96-well plates in a rigid position to enable sorting and temperature control. Cells were sorted directly into chilled BD Precise™ Single Cell Encoding 96-well plates containing lysis buffer, indexing dT primers and dNTPs. After sorting, plates were sealed with a foil cover, vortexed for 5–10 seconds, briefly centrifuged and frozen at −80 °C until further analysis. Precise plates were prepared for sequencing following manufacturer’s instructions for the BD Precise™ Reagents kit. Briefly, cDNA was synthesized, samples containing well-specific indices were pooled, and gene targets were amplified using 20 cycles and a custom primer panel. Plate indexes were added during the library amplification stage and plate pools were quantitated using the Agilent Bioanalyzer High Sensitivity DNA kit. Two nM dilutions from each plate were pooled, 1.44 pM libraries were loaded onto the Illumina MiSeq with 30% PhiX, and 2 × 75 bp reads were sequenced. Reads were mapped, assigned to Molecular Indexes (MI), and corrected using the Bowtie v2 aligner-based BD Precise™ Targeted Analysis Pipeline. (MI) counts were subjected to quality checks before downstream analysis. Wells with less than 50 total MI were rejected as poor-quality cells.
