# Supplementary material for: Circulating tumour cells are a prognostic indicator in advanced high-grade serous ovarian cancer and are associated with platelets and immune cells following dissemination
Source: Br J Cancer. 2025 Oct 10;134(1):22–32. doi: 10.1038/s41416-025-03227-7 (PMC12764790; doi:10.1038/s41416-025-03227-7)
Supplement: Supplementary file 3 — Supplemental Figures [file 41416_2025_3227_MOESM3_ESM.docx]

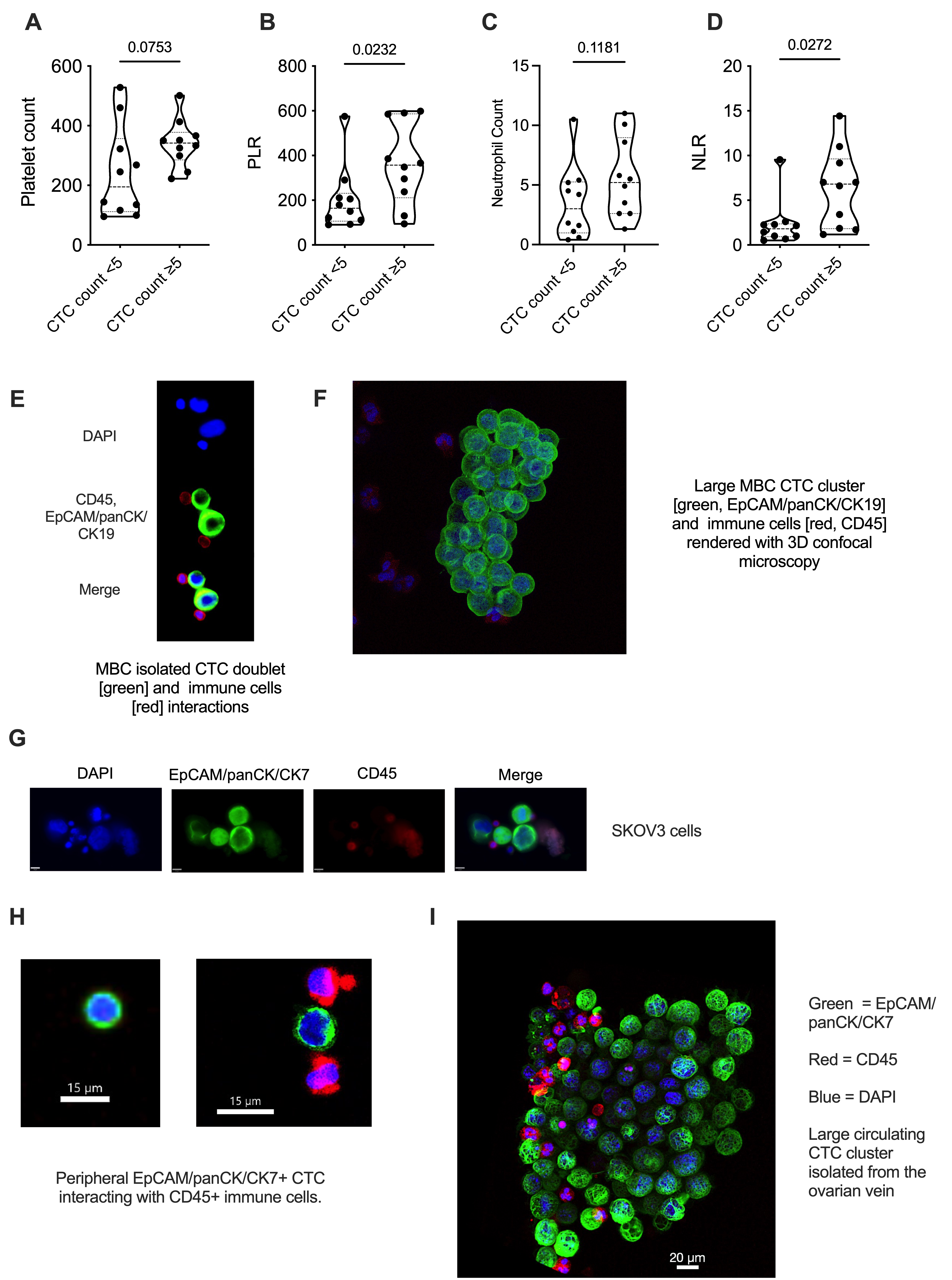


**Figure S1: Metastatic breast cancer patients with high levels of platelets have increased PLR and NLR counts.**

(A) Platelet count of MBC patients stratified into CTC counts <5 and CTC ≥ 5 CTCs detected in 7.5 ml blood by Parsortix enrichment (n=20). (B) PLR ratio of MBC patients stratified into CTC counts <5 and CTC ≥ 5 CTCs detected in 7.5 ml blood by Parsortix enrichment (n=20). (C) Neutrophil count and (D) NLR ratio of MBC patients stratified into CTC counts <5 and CTC ≥ 5 CTCs detected in 7.5 ml blood by Parsortix enrichment (n=20). (E) Representative image of CTC doublet with immune cells isolated from patient with MBC. (F) 3D confocal microscopy of large circulating CTC cluster or circulating tumour emboli isolated from patient with MBC using Parsortix. (G) SKOV3 cells (200 cells) were spiked into healthy donor blood (n=3) and stained with nuclear marker DAPI (blue), epithelial marker EpCAM/panCK/CK7 (green) and immune cell marker CD45 (red). (H) Example of typical peripheral HGSC ovarian CTC identified in 7.5ml using Parsortix and example of a rarer CTC-immune cell interaction identified in HGSC patient. (I) Example of a large CTC cluster (>40 cells) identified from the ovarian vein from patient with suspected HGSC. Large CTC cluster was infiltrated with immune cells. Patient was excluded as definitive diagnosis of HGSC could not be made with a diagnosis of endometrial endometrioid carcinoma suspected.

**
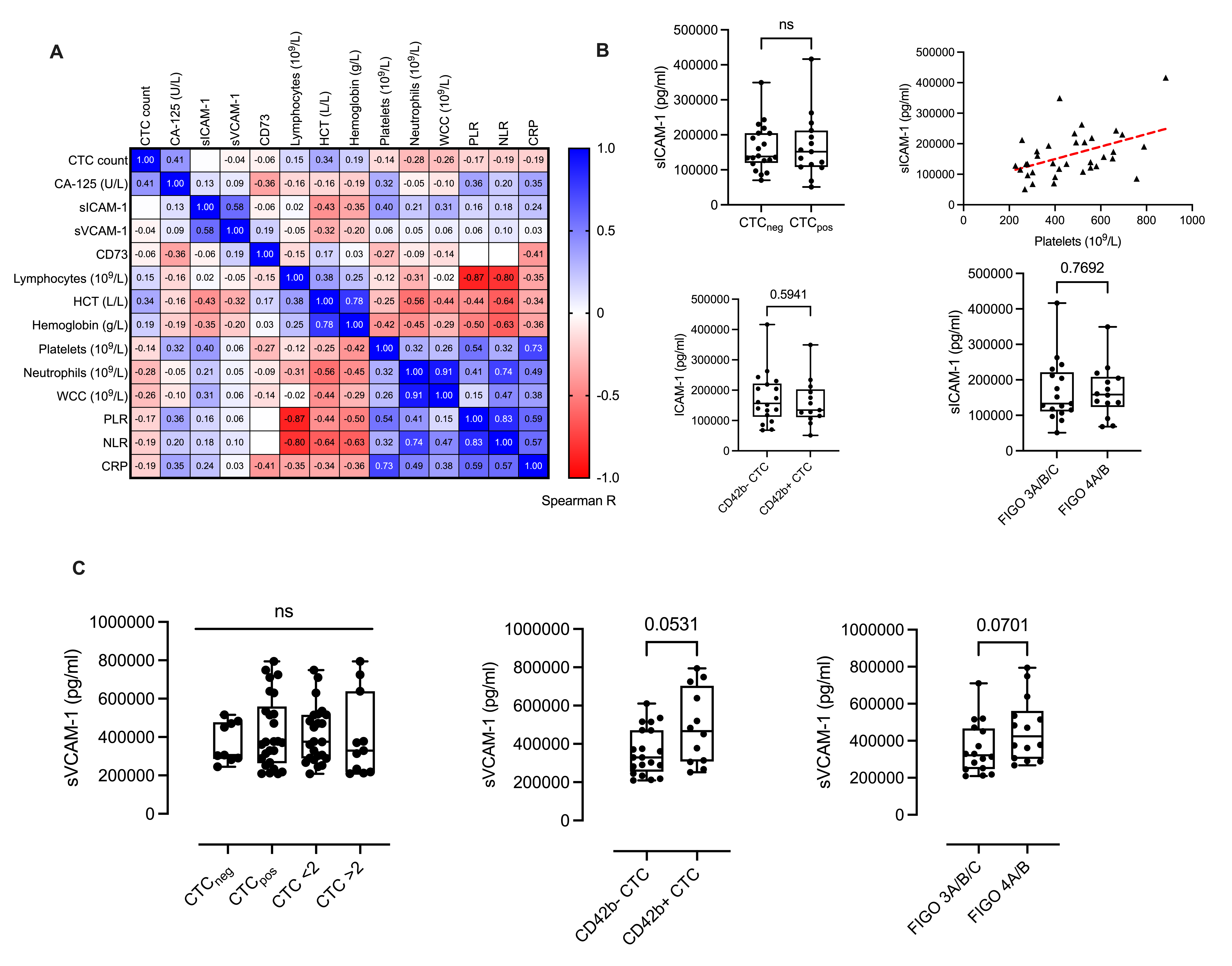
**

**Figure S2. Association of blood markers and markers of CTC vascular adhesion with CTC counts in HGSC.**

(A) Correlation matrix of cancer markers and standard immune/coagulation/haematological markers in HGSC patient cohort (n=34). (B) Levels of serum soluble ICAM-1 (sICAM-1) in CTCneg and CTCpos HGSC patients and correlation of sICAM-1 expression with platelet count. sICAM-1 expression was not altered between CD42+ and CD42- CTCs or FIGO staging in HGSC(n=37). (C) Expression of sVCAM-1 in CTC negative, CTC positive, CTC <2 and CTC ≥2 (n=38). * Indicates P<0.05.

**Figure S3: Gating strategies of enriched ovarian CTCs for single cell
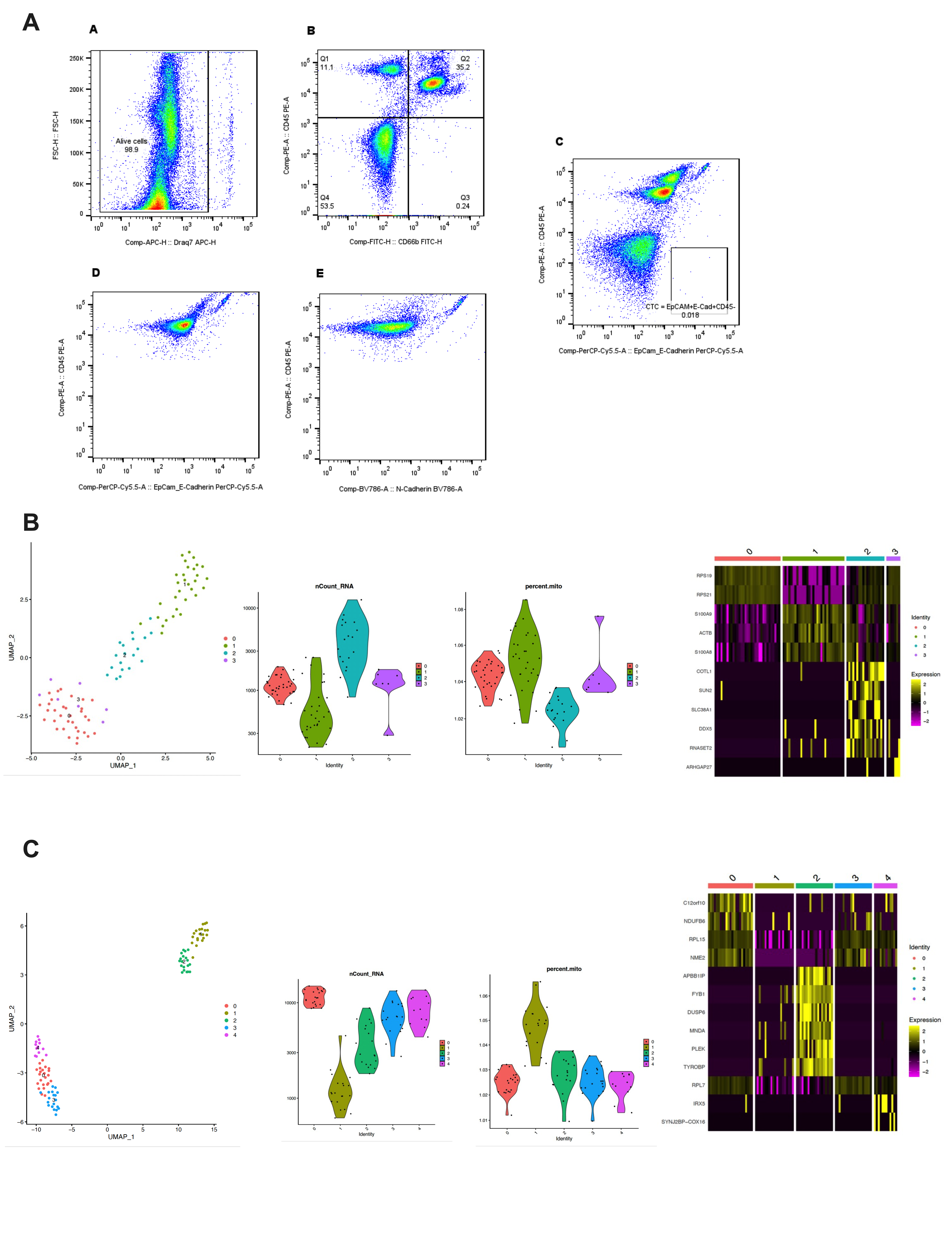
RNA sequencing.** (A) Gating strategies used for the establishment of single cell sorting of enriched CTCs from the ovarian vein using ClearCell FX. A CTC was defined as CD45 negative, EpCAM/E-Cadherin+. The population of cells positive for this was <1% at 0.018%. (B) PCA plots, nCount of number of RNA transcripts and percentage mitochondrial genes detected in the different populations of cells identified in ovarian vein sample. (C) PCA plots, nCount of number of RNA transcripts and percentage mitochondrial genes detected in the different populations of cells identified in breast cancer cell line control (MCF-7).

**
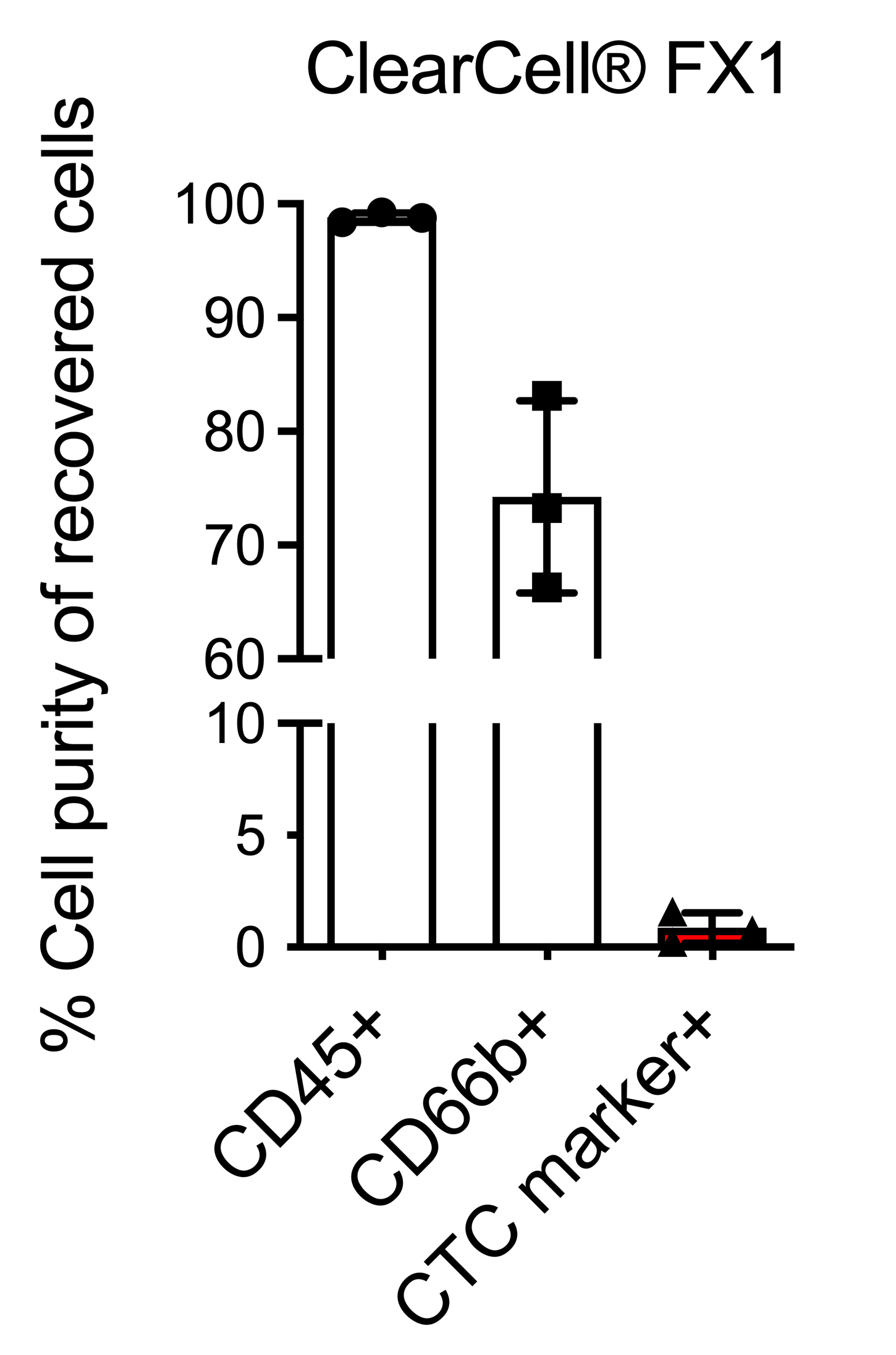

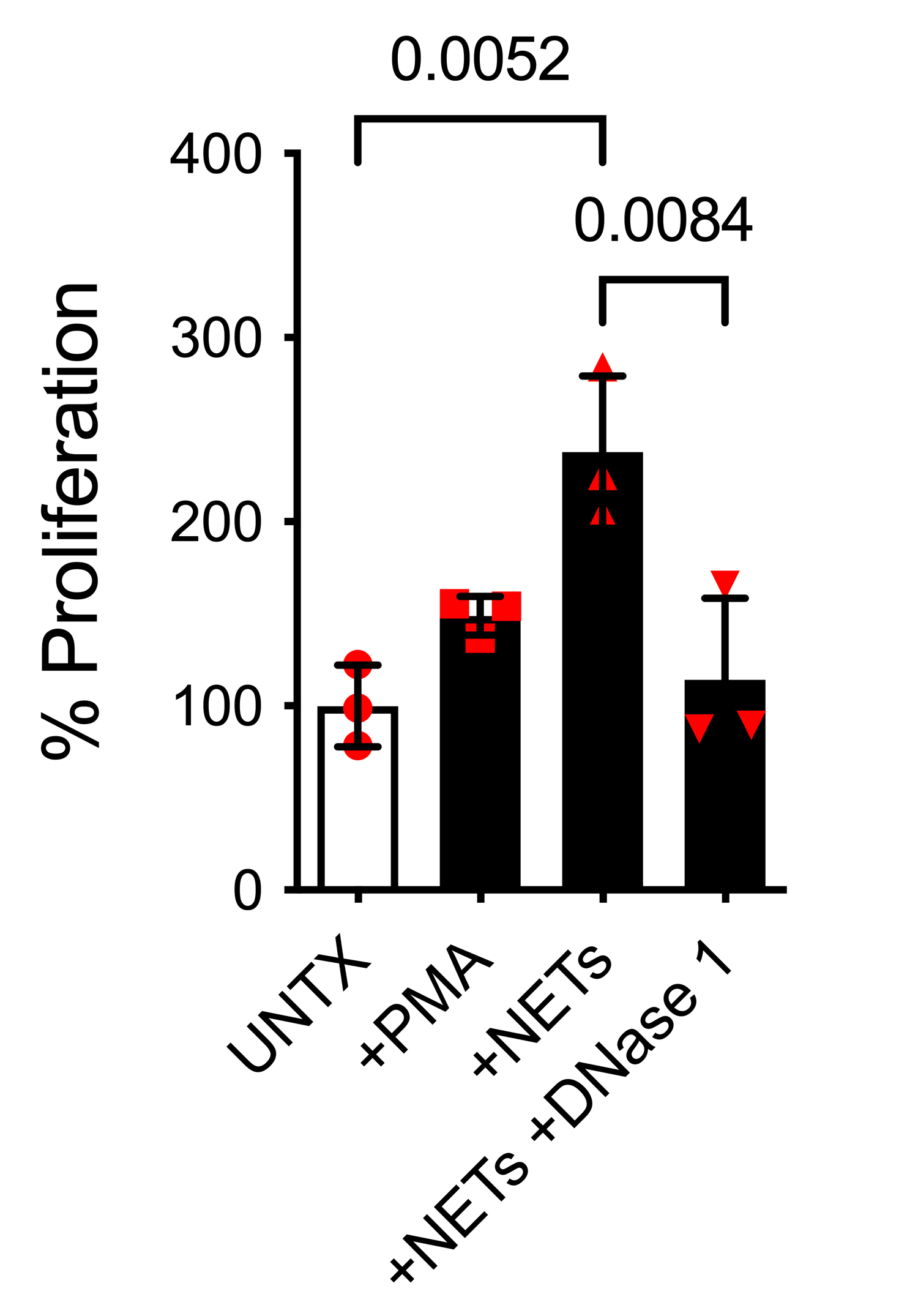
**

**Figure S4: Recovery and purity of ovarian vein isolated cells using ClearCell Fx enrichment.**

(A) Cells isolated from ClearCell FX from the ovarian vein were predominately CD45+ cells, with approximately 70% of CD45+ cells also staining for the neutrophil marker CD66. <1% of total number of cells enriched were positive for the CTC markers EpCAM/E-Cadherin (n=3). (B) Effect of co-culture of healthy donor neutrophils on ovarian cell line, SKOV3. Stimulation of neutrophil NETosis using 10 nM PMA promoted the proliferation of ovarian cancer cells, while pre-treatment with DNAse 1 negated the proliferative effects of the PMA induction of healthy donor NETosis (n=3).


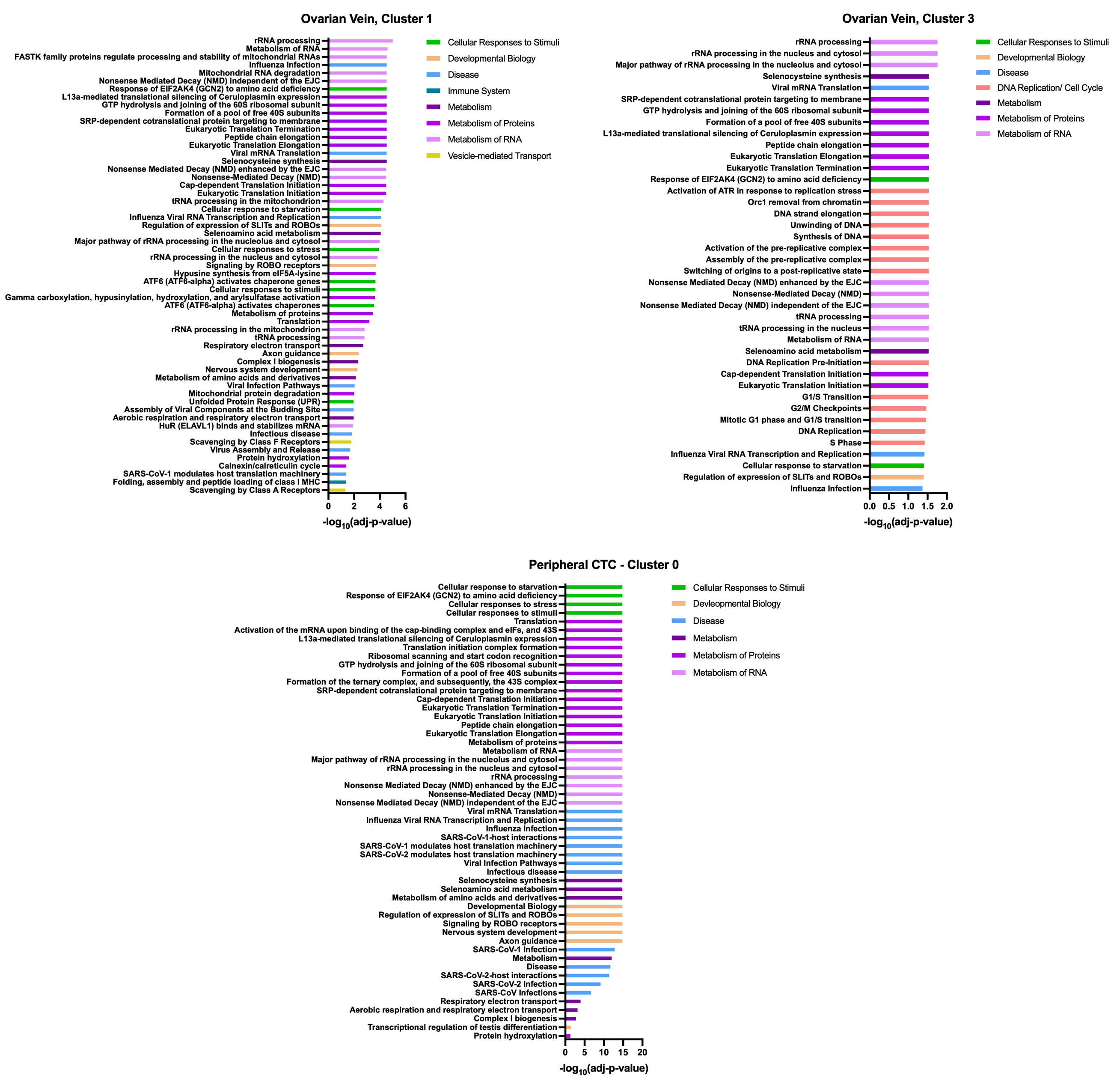


**Figure S5: Reactome mapping of scRNAseq data from CTCs enriched from the ovarian vein and peripheral blood.**
