# Supplementary material for: Circulating tumour cells are a prognostic indicator in advanced high-grade serous ovarian cancer and are associated with platelets and immune cells following dissemination
Source: Br J Cancer. 2025 Oct 10;134(1):22–32. doi: 10.1038/s41416-025-03227-7 (PMC12764790; doi:10.1038/s41416-025-03227-7)
Supplement: Supplementary file 4 — Supplemental Table 1 [file 41416_2025_3227_MOESM4_ESM.docx]

**Variable All Patients (n=20)**

**Median age** 53

Range (40-77)

**Median BMI 24.5**

Range (20-36)

**Histological Status**

ER+ 19 (95%)

PR+ 14 (70%)

HER2+ (FISH IHC positive) 2 (10%)

**Supplemental Table 1**: Clinicopathological Details of Metastatic Breast Cancer Cohort
