# Supplementary material for: Circulating tumour cells are a prognostic indicator in advanced high-grade serous ovarian cancer and are associated with platelets and immune cells following dissemination
Source: Br J Cancer. 2025 Oct 10;134(1):22–32. doi: 10.1038/s41416-025-03227-7 (PMC12764790; doi:10.1038/s41416-025-03227-7)
Supplement: Supplementary file 6 — Video 1 [file 41416_2025_3227_MOESM6_ESM.docx]

**Video 1: 3D confocal of CTC cluster isolated from the ovarian vein.**

IMARIS 9 software generated 3D structure of ovarian vein isolated CTC cluster interacting with CD45+ immune cell. CTC cluster was stained with DAPI (nuclear; blue), CD45 (immune marker; red) and EpCAM/panCK/CK7 (epithelial cell marker; green).
